# Association of Immune Related Adverse Events With Efficacy of Immune Checkpoint Inhibitors and Overall Survival in Cancers: A Systemic Review and Meta-analysis

**DOI:** 10.3389/fonc.2021.633032

**Published:** 2021-04-12

**Authors:** Yong Fan, Wenhui Xie, Hong Huang, Yunxia Wang, Guangtao Li, Yan Geng, Yanjie Hao, Zhuoli Zhang

**Affiliations:** ^1^ Department of Rheumatology and Clinical Immunology, Peking University First Hospital, Beijing, China; ^2^ Department of Respiratory and Critical Care Medicine, Peking University First Hospital, Beijing, China

**Keywords:** immune checkpoint inhibitors, immune-related adverse events, efficacy, cancer, meta-analysis

## Abstract

**Objectives:**

Immune checkpoint inhibitors (ICIs) have brought impressive benefits to cancer patients, however often accompanied with immune-related adverse events (irAEs). We aimed to investigate the association of irAEs with efficacy and overall survival in cancer patients treated by ICIs, and further quantify the association by stratifying subgroups.

**Methods:**

PubMed, EMBASE and Cochrane library from database inception to 29 August 2019 were systematically searched. Articles reporting association of objective response rate (ORR), progression-free survival (PFS), overall survival (OS) with irAEs in cancer patients treated with approved ICIs were included. Adjusted odds ratios (OR) with 95% confidential intervals (CIs) were calculated for ORR, and hazard ratios (HR) were used for PFS and OS.

**Results:**

A total of 52 articles comprising 9,156 patients were included. Pooled data demonstrated a statistically significant greater probability of achieving objective tumor response for patients with irAEs compared to those without (OR 3.91, 95% CI 3.05–5.02). In overall meta-analysis, patients who developed irAEs presented a prolonged PFS (HR 0.54; 95% CI 0.46–0.62) and OS (HR 0.51; 95% CI 0.41–0.59). More specifically, irAEs in certain cancer types (NSCLC and melanoma) and organs (skin and endocrine) were robustly associated with better clinical outcomes, while this association needs further verification regarding other tumors. High grade toxicities (G3–5) were not associated with a significantly favorable PFS or OS. Additionally, the association between irAEs and clinical benefit seemed to be more definite in patients receiving PD-(L)1 blockade than CTLA-4 blockade. Pooled data from landmark analyses displayed consistent results.

**Conclusions:**

The occurrence of irAEs predicted improved tumor response and better survival in overall cancer patients treated with ICIs. Notably, the association stayed robust in certain cancer types (NSCLC and melanoma) and organ-specific irAEs (skin and endocrine).

## Background

With the recent tremendous advances in cancer immunotherapy, the use of immune checkpoint inhibitors (ICIs) has brought remarkable benefit to patients with variable cancers (1, 2). Notably, ICIs are increasingly considered as the “fifth pillar” of cancer therapy, joining the ranks of surgery, cytotoxic chemotherapy, radiation, and targeted therapy. Furthermore, the list of indications for ICIs has also been extended, even as a first-line therapy (3, 4). Immune checkpoints, like cytotoxic T-lymphocyte antigen 4 (CTLA-4) and programmed cell death 1 (PD-1) or its ligand, programmed cell death ligand 1 (PD-L1), play key roles in immune homeostasis by controlling immune responses, maintaining self-tolerance and preventing autoimmunity. CTLA-4 is upregulated on T cell surface and competes with CD28 for binding to B7-1 (CD80) and B7-2 (CD86) on antigen presenting cells (5). In contrast to CD28 which is a costimulatory factor on T cells, CTLA-4 inhibits further activation of effector T cells. PD-1 is also an important negative regulatory receptor expressed on various immune cells, including T cells, B cells, and NK cells, and binds to its ligands PD-L1 (expressed widely in multiple tissues and tumor cells) and PD-L2 (restricted to professional antigen-presenting cells) (6, 7). PD-1 is mainly present on non-lymphoid cells in peripheral tissues; it generates local tolerance by dephosphorylating the T-cell receptor, leading to T-cell exhaustion (8). Antibodies against these immune checkpoints can directly release negative immune regulation of checkpoint and reactivate anti-tumor effect of cytotoxic T cells (9). Nevertheless, as a result of a highly active immune response, ICIs may lead to immune toxicities, known as immune-related adverse events (irAEs). In general, irAEs can develop in any organ/system at any time during ICIs treatment or even after cessation of ICIs (1, 6). However, most of irAEs happen within weeks to 3 months after initiation of immune therapy. The majority of irAEs are mild to moderate and the frequency differs across ICI types. A comprehensive systematic analysis revealed that the overall AEs occurred in 74% cancer patients treated with PD-(L)1 inhibitors, 89% in CTLA-4 inhibitor group and up to 90% in ICIs combination group. Severe irAEs (≥grade 3) were reported in 14% patients treated with PD-(L)1 inhibitors, 34% patients treated with CTLA-4 inhibitor, and 55% patients with ICIs combinations (10). Patterns of irAEs also differ per ICI treatment. Certain irAEs like rash, colitis, and hypophysitis are more common with CTLA-4 blockade, while pneumonitis and hypothyroidism are more frequently with PD-1 blockade (11).

Although the precise pathophysiology of irAEs remains unclear, the occurrence of irAEs may represent the reinvigoration of immune system to some extent. Accordingly, it has been hypothesized that certain patients who experienced irAEs would have affirmative enhancement of immune response with better response to ICIs. But a study with a large sample size failed to show the association of irAEs with clinical outcomes (12). On the other hand, a very recent meta-analysis from Petrelli et al. demonstrated a positive association between irAEs and efficacy of ICIs (13), however limited approved immunotherapeutic agents were included in the analysis. So far, it is still unclear whether there is an association between irAEs with efficacy and overall survival in those cancer patients who receive ICIs therapy. If the association exists, whether the association will be affected by specific cancer types, ICIs strategies, organ specific-irAEs, or different geographical regions also needs to be explored. Importantly, a large amount of high-quality studies have emerged after Petrelli’s review. Therefore, we performed an updated and comprehensive systematic review and meta-analysis, aiming to provide better understanding and enhance the insight on individualized therapy to improve outcomes in cancer patients, stratified by cancer types, ICIs types, organ specific-irAEs, and geographical regions. Landmark analysis was also conducted in the present study.

## Methods

This systematic review and meta-analysis were conducted by following the Preferred Reporting Items for Systematic Reviews and Meta-analyses (PRISMA) guidelines (14).

### Search Strategies and Selection Criteria

Databases of PubMed, EMBASE, and the Cochrane Library were searched to identify the relevant articles from the inception to Aug 29, 2019 by three independent authors (YF, WHX and HH). The following search terms were used: (1) Term 1: immune checkpoint inhibitor OR checkpoint blockade OR CTLA-4 OR cytotoxic T lymphocyte associated protein 4 OR CTLA-4 Inhibitor OR PD-1 OR programmed death receptor 1 OR PD-1 Inhibitor OR PD-L1 OR Programmed death-ligand 1 OR PD-L1 Inhibitor OR ipilimumab OR YERVOY OR nivolumab OR OPDIVO OR pembrolizumab OR KEYTRUDA OR cemiplimab OR LIBTAYO OR atezolizumab OR TECENTRIQ OR avelumab OR BAVENCIO OR durvalumab OR IMFINZI; (2) Term 2: adverse event OR immune related adverse event OR irAE OR toxicity; (3) Term 3: case control OR observational OR trial OR clinical study OR intervention study OR retrospective OR prospective OR cohort study; (4) Term 4: cancer OR malignancy OR tumor OR neoplasm OR carcinoma OR melanoma OR NSCLC OR leukemia OR lymphoma; (5) Term 5: efficacy OR response OR prognosis OR survival OR benefit. No restriction for publication year, while language was restricted to English. Additional records were procured by a hand-search of the references of primitive literature, and reviews were also identified so as to not miss any eligible studies.

The studies met the following inclusion criteria were eligible for the meta-analysis: (1) studies assessing inhibitors of PD-(L)1, CTLA-4, or both in cancer patients (only seven ICIs approved by the US Food and Drug Administration till Aug, 2019); (2) studies with data available for detailed events number who achieved objective tumor response, or provided the adjusted odd ratio (OR) for objective response rate (ORR) and/or the adjusted hazard ratio (HR) for Progression Free Survival (PFS) and/or the adjusted HR for Overall Survival (OS) based upon irAEs presence; (3) The most informative or recent publication was selected in case of studies from the same institution with similar or duplicated subjects. Studies were excluded if (1) no available data of HR even if survival time was provided in both groups; (2) case reports or case series with sample size of <15 patients; (3) reviews.

### Data Extraction and Quality Assessment

Data were extracted by three authors (YF, WX and HH) from the identified studies in duplicate using a common extraction form. Any disagreement was resolved by the third experienced reviewer (ZZ). We extracted the following information from the included studies: name of first author, publication year, study design, study period, country, cancer types, ICIs types; number of patients with ICIs; gender; median age (years); number of irAEs; number of non-irAEs; number of patients who achieved therapeutic response (overall response and/or complete and/or partial response), OR with 95% CI for ORR and/or HR with 95% CI for PFS and OS. We contacted authors for additional information of study when necessary.

The quality of selected studies was appraised using the Newcastle-Ottawa quality assessment scale (NOS) (15). Each study was accordingly assessed in three main areas: patient selection (0–4 points), comparison between study groups (0–2 points), and assessment of exposure (0–3 points). The quality of the eligible studies was independently assessed by two investigators (YF and WX), and disagreements were resolved by reaching consensus. A study with scale > 6 was regarded as high-quality.

### Statistical Analysis

The primary endpoint was ORR. The secondary endpoints included PFS and OS. We also pooled the results of landmark analysis in available studies to confirm the main findings. Furthermore, a series of subgroup analyses were conducted to explore the variations of the effect of cancer types, ICIs types, ethnics (regional distribution) and organ specific-irAEs on ICIs efficacy in tumor. Subgroups analysis was only performed when at least two studies were available.

Study heterogeneity was evaluated by I^2^ index. For a study with I^2^ >50% indicating significant heterogeneity, a random-effect model was used for the analysis; otherwise, a fixed-effect model was applied. Pooled OR with 95% CI was calculated to assess the association of irAEs occurrence with therapeutic response, and a value >1 indicated favorable response in tumor to presence of irAEs. Meanwhile, pooled HR with 95% CI was measured to evaluate the association between irAEs occurrence and cancer PFS and OS, and HR <1 with no overlapping 95% CI was considered as having statistically significant positive association. In addition, sensitivity analyses were conducted by excluding each individual study to assess its influence on the overall risk. Publication bias was assessed by visualization of funnel plot, Egger and Begg tests. All reported P values were 2-sided, and P <0.05 was used to indicate statistical significance. All analyses were performed using STATA software (version 11.0, Stata Corp.).

## Results

### Results of Literature Search and Characteristics of Identified Studies

**Figure 1:**
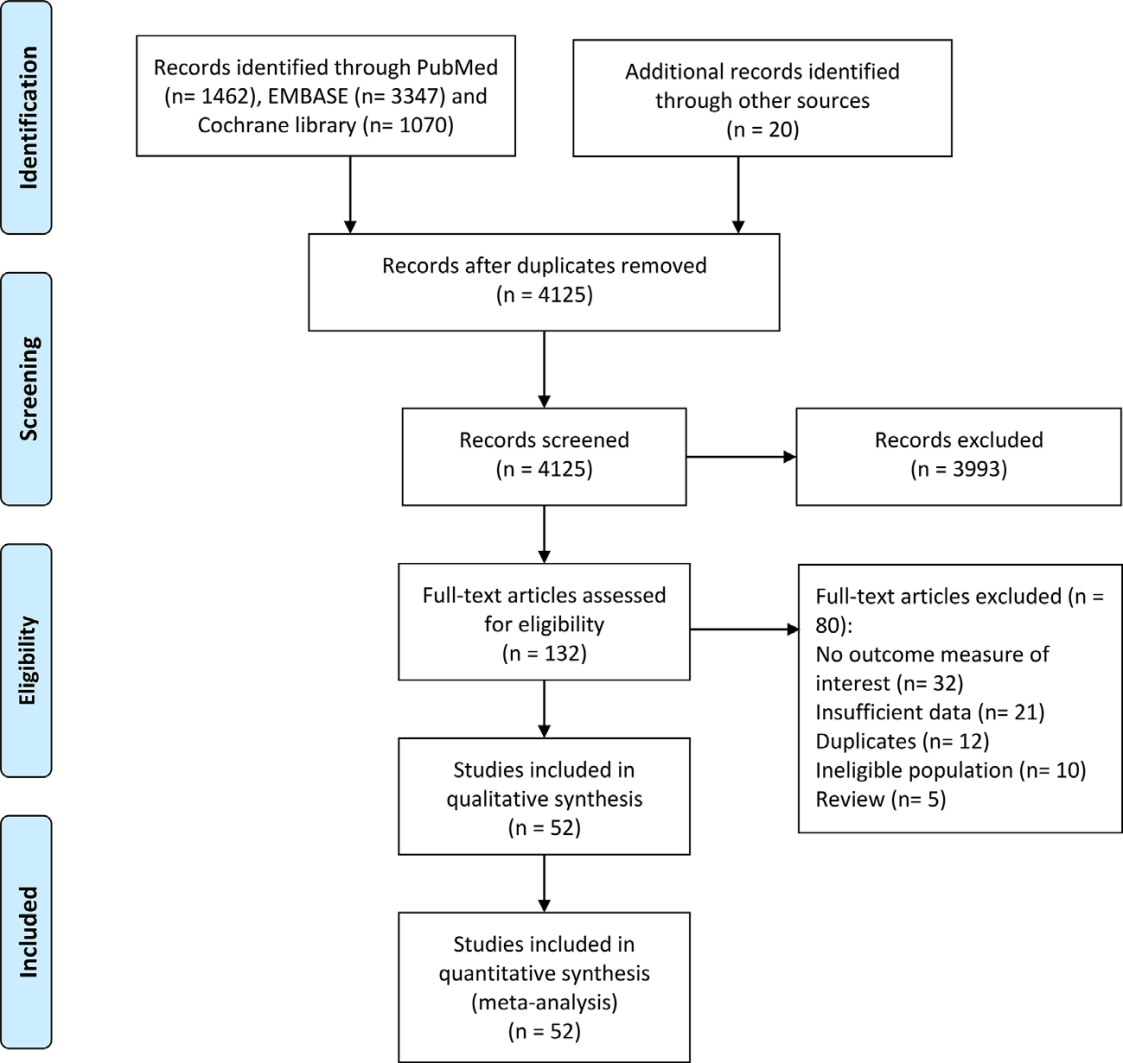
Flow chart of the article identification, inclusion, and exclusion.

Our initial search of database yielded 4,125 results. After a careful review of the titles and abstracts, 132 potentially eligible studies were subsequently taken to a full-text review for more detailed evaluation. In total, 52 articles comprising 9,156 patients met predefined criteria were included in this meta-analysis (12, 16–66). The details of search program in this study were shown in **Figure 1**.

All the included studies were retrospective (including seven studies extracted data from clinical trials), except eight prospective studies. Regarding the type of tumor, there were 18 studies in melanoma, 19 in non-small cell lung cancer (NSCLC), three in renal cell cancer, one in head and neck cancer, and the resting 11 studies in mixed cancers. As for the ICIs use, PD-1 inhibitors and/or PD-L1 inhibitors were investigated in most of the eligible studies, CTLA-4 inhibitors only in 10 studies and ICIs mixed/combined therapy in four studies. Geographically, 17 studies were from Asian-pacific countries, 15 from European countries, 18 from USA, one from Canada, and one study pooled data from international RCTs. The median number of patients enrolled in each study was 108 (ranged 15–855). **Table 1** listed the main characteristics of the 52 eligible studies.

**Table 1:** Characteristics and end-points (ORR, PFS, OS) of enrolled studies.

ID	First author	Publication year	Study design	Country, institution	Malignancy	ICIs types	Patients with ICIs	Number of irAEs (n, %)	Number of non-irAEs (n, %)	Organ specific irAEs	irAEs Response (n)	non-irAEs Response (n)	adjusted ORR (95%CI)	adjusted PFS (95%CI)	adjusted OS (95%CI)
1	Attia et al. (16)	2005	prospective	USA, National Cancer Institute (NCI)	Melanoma	CTLA-4	56	17	39	Mixed	5	2	/	/	/
2	Beck et al. (17)	2006	retrospective	USA, NCI	Melanoma + Renal Cell Carcinoma	CTLA-4	198	39	150	Enterocolitis	14	12	/	/	/
3	Downey et al. (18)	2007	Two trials	USA, NCI	Melanoma	CTLA-4	139	86	53	Mixed	22	1	/	/	/
4	Yang et al. (19)	2007	phase II trial	USA, NCI	Renal Cell Cancer	CTLA-4	61	20	41	Mixed	6	0	/	/	/
5	Weber et al. (20)	2008	Phase I/II RCT study	USA, multicenters	Melanoma	CTLA-4	88	63	25	Mixed	4	0	/	/	/
6	Ku GY et al. (21)	2010	retrospective	USA, Memorial Sloan-Kettering Cancer Center	Melanoma	CTLA-4	53	15	36	Mixed	4	2	/	/	/
7	Ascierto et al. (22)	2014	retrospective	Italy, EAP study	Melanoma	CTLA-4	855	278	555	Mixed	41	70	/	/	/
8	Horvat et al. (12)	2015	retrospective	USA, Memorial Sloan Kettering Cancer Center	Melanoma	CTLA-4	298	254	44	Mixed	/	/	/	0.93(0.66,1.31)	0.95(0.61,1.46)
9	Sanlorenzo et al. (23)	2015	retrospective	USA, Mt Zion Cancer Research Center	Mixed	PD-1	83	35	48	Skin	/	/	/	0.82 (0.17–4.06)^a^; 0.70 (0.05–9.50)^b^; 0.12 (0.02–0.74)^c^	/
10	Dick et al. (24)	2016	retrospective	Germany, NCT	Melanoma	CTLA-4	86	36	50	Mixed	13	3	13.85(2.64,72.69)	0.45(0.22,0.92)	0.45(0.24,0.86)
11	Freeman et al. (25)	2016	retrospective analysis of two phase I studies	USA, Moffitt Cancer Center	Melanoma	PD-1	148	101	47	Mixed	/	/	data for skin, GI, endocrine, lung		
12	Hasan et al. (26)	2016	retrospective	Switzerland, Cantonal Hospital St. Gallen	NSCLC	PD-1	40	7	33	Skin	5	7	/	/	/
13	Hua et al. (27)	2016	prospective	France, Gustave Roussy Institute	Melanoma	PD-1	67	17	50	vitiligo	12	14	/	/	/
14	Nakamura et al. (28)	2016	retrospective	Japan, Keio University Hospital	Melanoma	PD-1	98	51	47	Mixed	/	/	/	/	0.54(0.3,0.99)
15	Judd J et al. (29)	2017	retrospective	USA, Fox Chase Cancer Center	Mixed	PD-1	160	64	96	Mixed	16	12	3.027 (1.220-7.734) low-grade; 1.454 (0.339-6.235) high-grade	0.79 (0.45–1.38) low-grade; 0,87 (0.49–1.55) high-grade	0.67 (0.37–1.23) low-grade; 1.00 (0.45–2.20) high-grade
16	Kim et al. (30)	2017	prospective	Korea, two centers	NSCLC	PD-1	58	19	39	Thyroid	/	/	/	0.38(0.17,0.85)	0.11(0.01,0.92)
17	Osorio et al. (31)	2017	retrospective analysis of KEYNOTE-1	USA, Memorial Sloan Kettering Cancer Center	NSCLC	PD-1	51	10	41	Thyroid	/	/	/	0.58(0.27,1.21)	0.29(0.09,0.94)
18	Teraoka et al. (32)	2017	prospective	Japan, Kobe City Medical Center	NSCLC	PD-1	43	27	16	Mixed	9	2	/	/	/
19	Weber et al. (33)	2017	retrospective	International multi-centers	Melanoma	PD-1	576	255	321	Mixed	124	57	/	/	/
20	Yamazaki et al. (34)	2017	phase II trial	Japan, multicenters	Melanoma	PD-1	24	20	4	Mixed	/	/	/	0.13(0.05,0.37)	0.1(0.03,0.36)
21	Faje et al. (35)	2018	retrospective	USA, Massachusetts General Hospital	Melanoma	CTLA-4	281	64	217	hypophysitis	/	/	/	/	0.53(0.36,0.75)
22	Fujimoto et al. (36)	2018	retrospective	Japan, multicenters	NSCLC	PD-1	613	/	/	Mixed (Grades 3–5)	/	/	/	0.76(0.55,1.01)	/
23	Fujisawa et al. (37)	2018	retrospective	Japan, multicenters	Melanoma	CTLA-4 after PD-1	60	47	23	Mixed	/	/	data for skin, endocrine		
24	Haratani et al. (38)	2018	retrospective	Japan, multicenters	NSCLC	PD-1	134	44	61	Mixed	23	17	/	0.542(0.295,0.971)	0.285(0.102,0.675)
25	Kostine et al. (39)	2018	prospective	France, Centre Hospitalier Universitaire	Mixed	monotherapy or combined	524	156	368	Mixed	118	130	/	/	/
26	Lesueur et al. (40)	2018	retrospective	France, multicenters	NSCLC	PD-1	104	62	42	Mixed	/	/	/	0.66(0.433,1.099)	0.64(0.377,1.087)
27	Lisberg et al. (41)	2018	retrospective analysis of KEYNOTE-1	USA, UCLA	NSCLC	PD-1	97	39	58	Mixed	15	5	2.02(0.87,4.69)	0.75(0.56,0.99)	0.75(0.58,0.96)
28	Min Lee et al. (42)	2018	retrospective	USA, Stanford University	Mixed	PD-1/PD-L1	114	20	94	Dermatitis	13	16	7.3(2.3,23.1)	/	/
29	Owen et al. (43)	2018	retrospective	USA, Wexner Medical Center	NSCLC	PD-1/PD-L1	91	27	64	Mixed	/	/	/	/	0.364(0.203,0.649)
30	Sato et al. (44)	2018	prospective	Japan, Wakayama Medical University	NSCLC	PD-1	38	11	27	Mixed	7	20	/	0.1(0.02,0.37)	/
31	Shafqat et al. (45)	2018	retrospective	USA, Medical University of South Carolina	Mixed	PD-1/PD-L1	157	42	115	Mixed	/	/	/	0.339(0.187,0.617)	/
32	Suh et al. (46)	2018	retrospective	Korea, SNUH and SNUBH	NSCLC	PD-1	54	12	42	Mixed	8	10	/	0.5(0.22,1.13)	0.48(0.2,1.14)
33	Toi et al. (47)	2018	retrospective	Japan, Sendai Kousei Hospital	NSCLC	PD-1	137	66	71	Mixed	34	9	/	0.45(0.3,0.68)	0.42(0.24,0.71)
34	Abu-Sbeih et al. (48)	2019	retrospective	USA, MD Anderson Cancer Center	Melanoma	monotherapy or combined	346	173	173	Diarrhea/colitis	/	/	/	0.56(0.41,0.76)	0.53(0.36,0.78)
35	Ahn et al. (49)	2019	retrospective	Korea, Yonsei Cancer center	NSCLC	PD-1	111	51	60	Mixed	21	16	/	0.434(0.256,0.735)	0.484(0.255,0.919)
36	Berner et al. (50)	2019	prospective	Switzerland multicenters	NSCLC	PD-1	73	48	25	skin	/	/	5.28(1.78,15.67)	0.22(0.09,0.49)	0.29(0.12,0.71)
37	Bjornhart et al. (51)	2019	retrospective	Denmark, University Hospital of Odense	NSCLC	Mixed ICIs	118	/	/	Mixed	/	/	/	0.71(0.39,1.27)	0.47(0.21,1.05)
38	Cortellini et al. (52)	2019	retrospective	Italy, multicenters	NSCLC	PD-1	559	215	292	Mixed	100	75	/	0.57(0.45,0.72)	0.47(0.36,0.6)
39	Grangeon et al. (53)	2019	retrospective	France, two centers	NSCLC	PD-1/PD-L1	270	124	146	Mixed	/	/	4.9(2.18,11.05)	0.42(0.32,0.57)	0.29(0.18,0.46)
40	Indini et al. (54)	2019	retrospective	Italy, Fondazione IRCCS Istituto	Melanoma	PD-1	173	102	71	Mixed	/	/	1.95(0.91,4.15)	0.47(0.26,0.86)	0.39(0.18,0.81)
41	Ishihara et al. (55)	2019	retrospective	Japan, Tokyo Women’s Medical University	Renal Cell Cancer	PD-1	47	23	24	Mixed	14	3	/	0.25(0.11,0.56)	/
42	Ksienski et al. (56)	2019	retrospective	Canada BC cancer centers	NSCLC	PD-1	271	116	155	Mixed	/	/	data for severity of irAEs		
43	Lang et al. (57)	2019	prospective	German, University ofHeidelberg	Melanoma	CTLA-4	100	49	51	Diarrhea	12	9	/	1.93(1.01,3.68)	1.57(0.75,3.29)
44	Lei et al. (58)	2019	retrospective	USA, Henry Ford Hospital	Mixed	PD-1	103	34	69	Thyroid	13	12	2.8(0.89,9.2)	0.45(0.27,0.76)	0.4(0.19,0.85)
45	Liew D et al. (59)	2019	retrospective	Australia, Olivia Newton-John Cancer	Mixed	PD-1	244	91	153	Rheumatic	/	/	11.16(2.65,46.98)	/	/
46	Okada et al. (60)	2019	retrospective	Japan, multicenters	Melanoma	PD-1	15	8	7	Mixed	2	0	/	/	0.01(<0.01,0.88)
47	Okamoto et al. (61)	2019	retrospective	Japan, multicenters	Head and neck cancer	PD-1	100	30	70	Mixed	6	7	/	/	/
48	Ricciuti et al. (62)	2019	retrospective	Italy, multicenters	NSCLC	PD-1	195	85	110	Mixed	37	11	/	0.48(0.34,0.67)	0.38(0.26,0.56)
49	Rogado et al. (63)	2019	retrospective	Spain, Hospital Universitario de la Princesa	Mixed	PD-1	106	40	66	Mixed	33	11	/	0.435(0.278,0.714)	0.909(0.625,1.429)
50	Sakakida et al. (64)	2019	retrospective	Japan, Kyoto Prefecture University	Mixed	PD-1	150	25	125	Thyroid	/	/	/	0.56(0.29,1.02)	0.42(0.16,0.97)
51	Verzoni et al. (65)	2019	retrospective	Italy, EAP study	Renal Cell Cancer	PD-1	389	76	313	Mixed	/	/	/	/	0.48(0.3,0.78)
52	Yamauchi et al. (66)	2019	retrospective	Japan Kyoto University Hospital	Mixed	PD-1	200	67	133	thyroid	/	/	/	0.66(0.46,0.95)	0.61(0.39,0.93)

^a-c^represents 3 different therapeutic regimens. a indicates cancer patients treated with 10 mg/kg of pembrolizumab every 3 weeks; b indicates cancer patients treated with 10 mg/kg of pembrolizumab every 2 weeks; c indicates cancer patients treated with 2mg/kg of pembrolizumab every 3 weeks.

Among the 8,372 patients included [demographic data were absent in four studies (25, 35, 49, 59)], 5,323 (63.6%) were male and 3,049 (36.4%) were female. The median age of patients was typically in the 60s (ranged from 50.0 to 71.5 years) across all studies, and median follow-up ranged from 3.0 to 26.2 months. In this meta-analysis, irAEs of any grade and grades 3–5 occurred in 39.9 and 11.0% of patients, respectively.

### Association of irAEs Occurrence With Higher Response Rate

**Figure 2:**
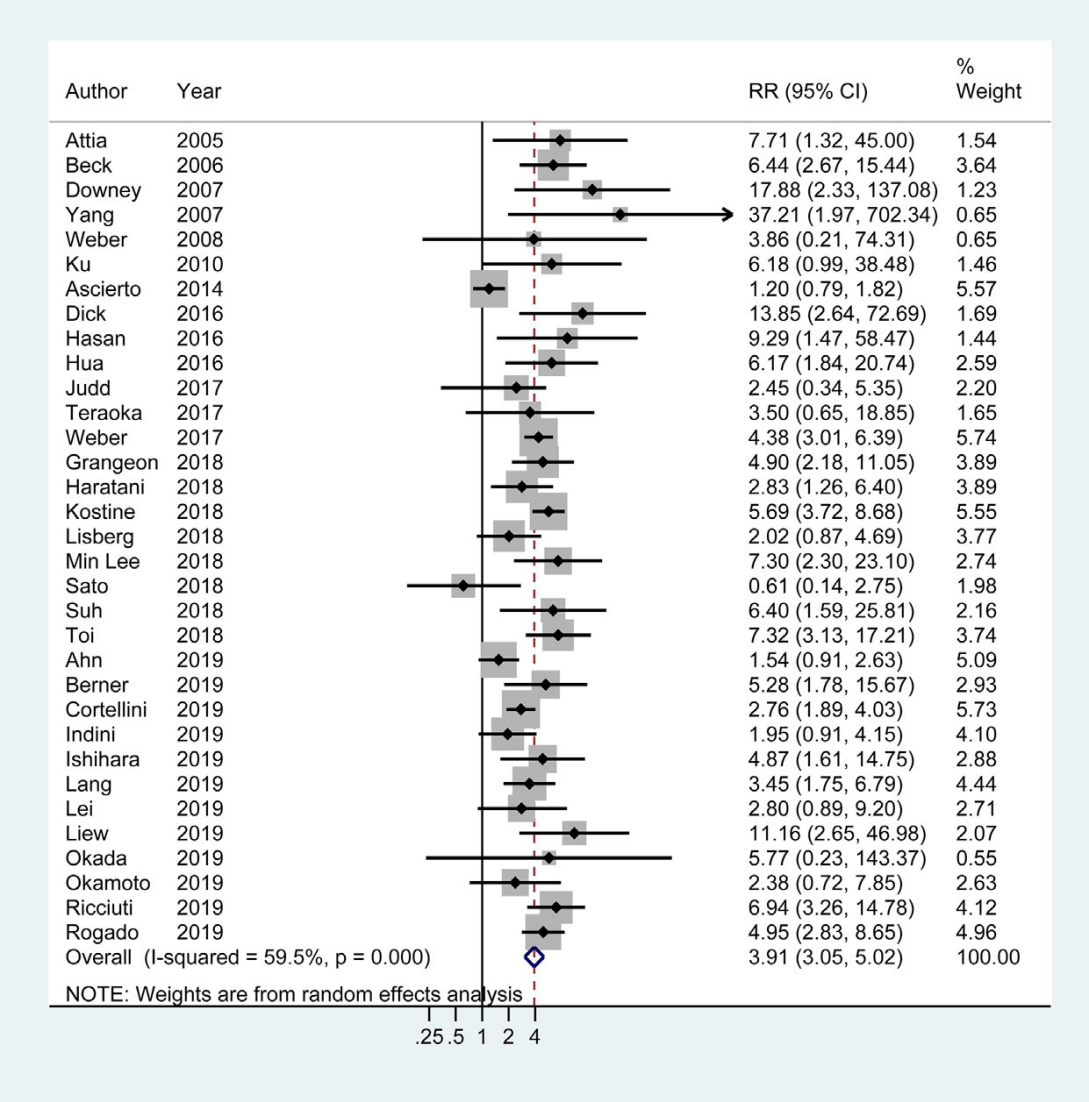
Forest plot of the association of immune-related adverse events with tumor objective response rate (ORR) in cancer patients receiving immune checkpoint inhibitors. RR, risk ratio; CI, confidence interval.

There were 33 articles (four only provided adjusted OR with 95% CI) with available data of objective response in tumor. Overall, objective treatment response was observed in 40.6% (731/1,807) patients with irAEs, in contrast to 18.2% (533/2,935) patients without irAEs. Meta-analysis demonstrated a nearly 3-fold higher probability of achieving objective response in tumor in patients with irAEs compared to those without (OR 3.91, 95% CI 3.05–5.02; I^2^ = 59.5%) (**Figure 2**).

### Association of irAEs Occurrence With Better PFS and OS

**Figure 3:**
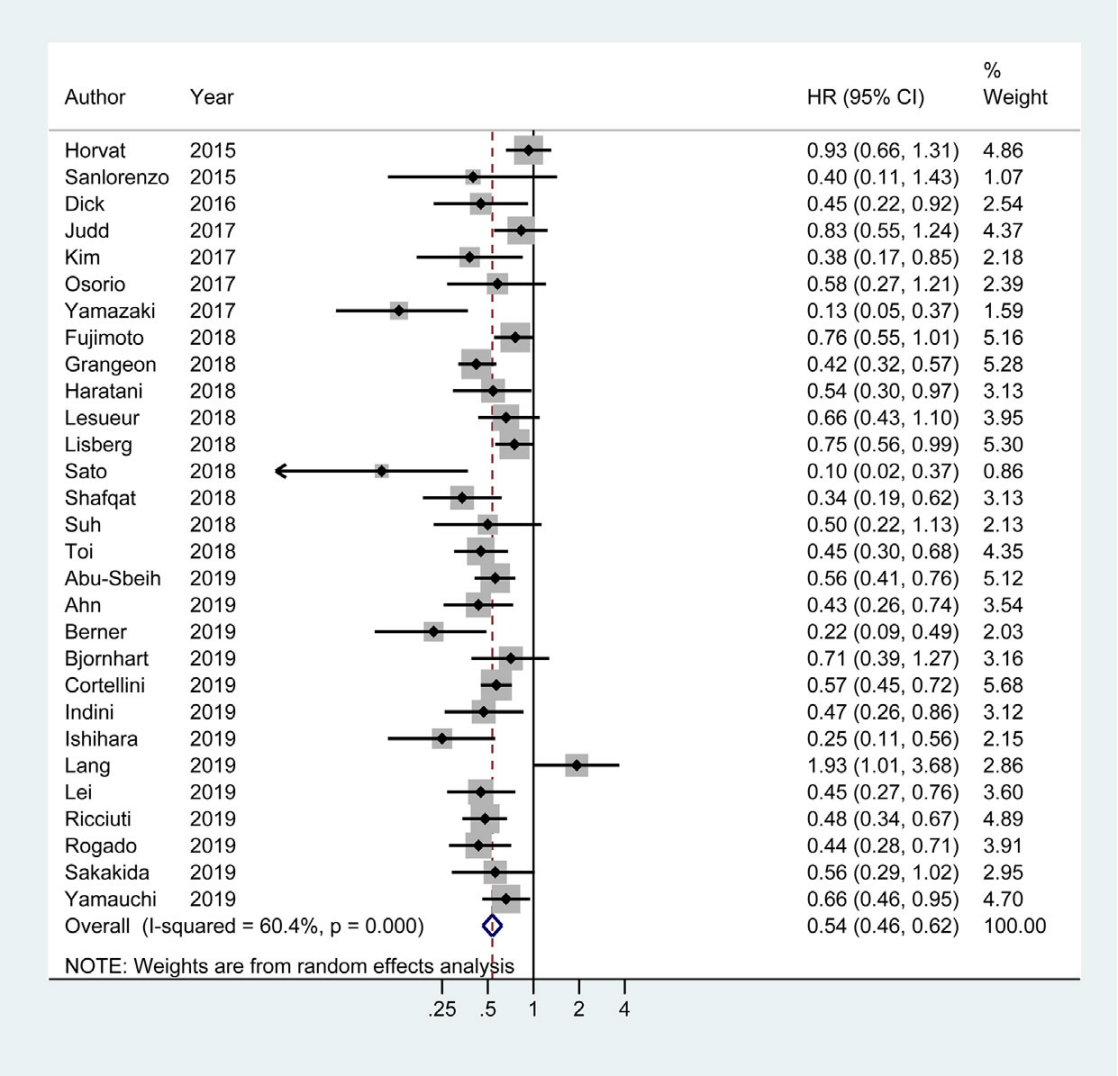
Forest plot of the association of immune-related adverse events with progression free survival (PFS) in cancer patients receiving immune checkpoint inhibitors. HR, hazard ratio; CI, confidence interval.

**Figure 4:**
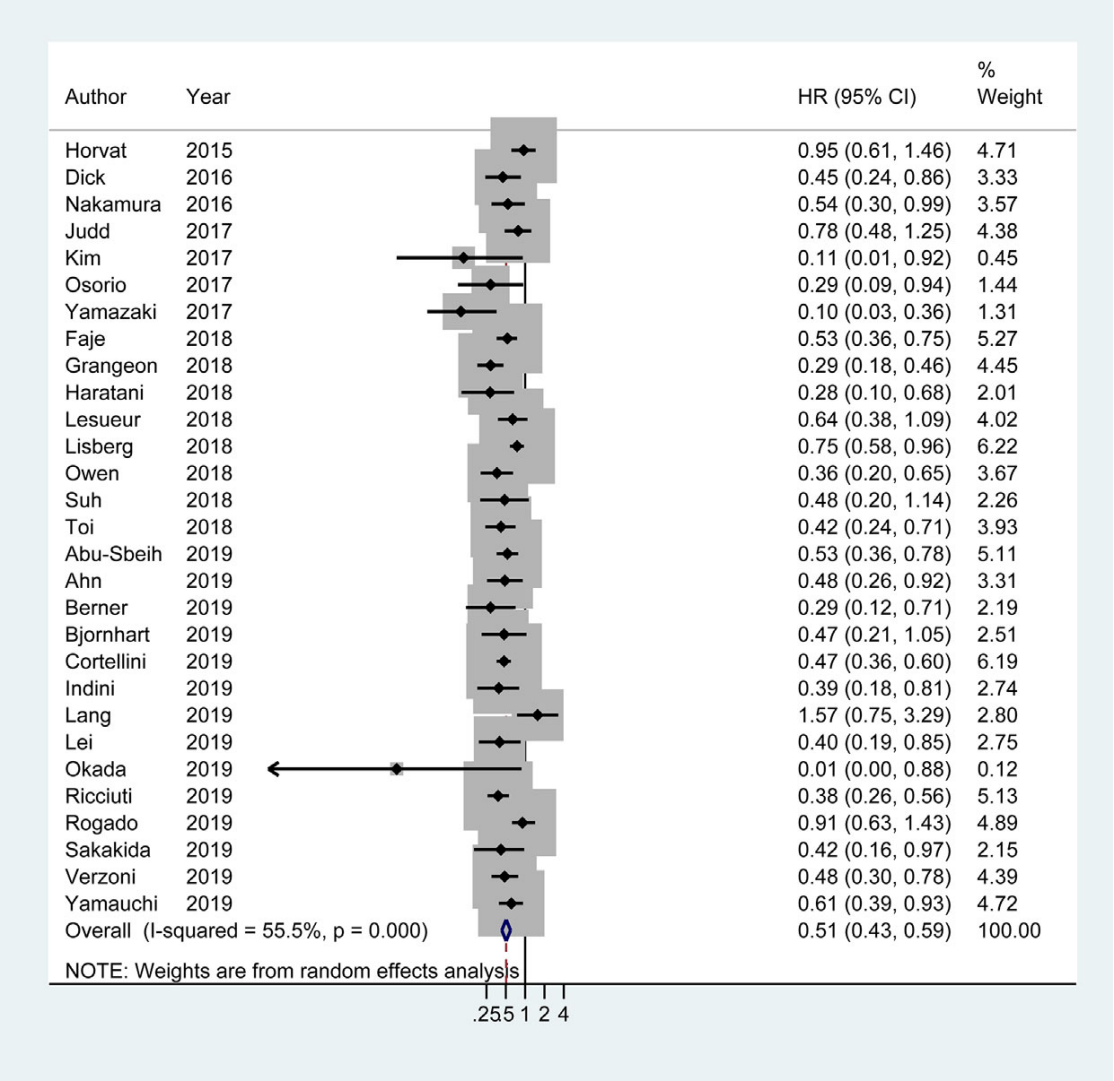
Forest plot of the association of immune-related adverse events with overall survival (OS) in cancer patients receiving immune checkpoint inhibitors. HR, hazard ratio; CI, confidence interval.

Of the 29 studies involving 4,645 patients available for PFS analysis, 19 studies showed a superior PFS in irAEs group while 10 studies showed no significant difference. A pooled analysis showed that patients experiencing irAEs had a better PFS of ICIs therapy than non-irAEs participants (HR 0.54; 95% CI 0.46–0.62; I^2^ = 60.4%) (**Figure 3**). For OS analysis, a total of 29 studies (4,581 patients) were included. Meta-analysis demonstrated that patients with irAEs had a significantly reduced risk of death compared to those without irAEs (pooled HR 0.51; 95% CI 0.41–0.59; I^2^ = 55.5%) (**Figure 4**).

### Landmark Analysis of irAEs With PFS and OS

**Figure 5:**
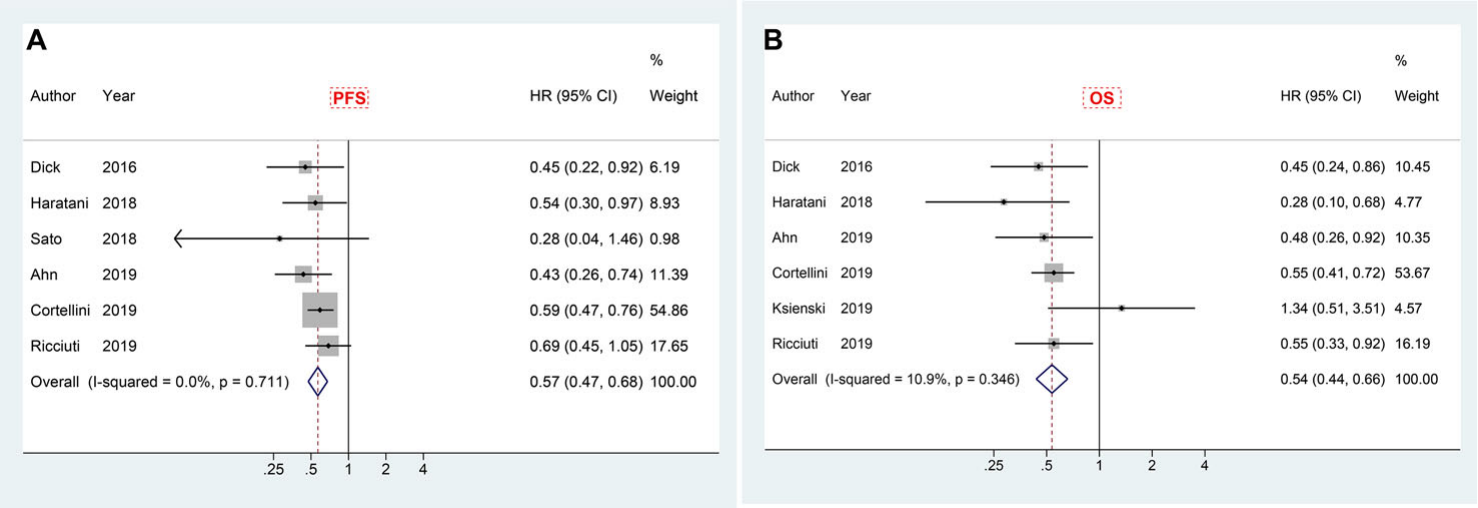
Forest plot of landmark analysis in exploring the associations of immune-related adverse events with PFS **(A)** and OS **(B)** in cancer patients receiving immune checkpoint inhibitors. HR, hazard ratio; CI, confidence interval; PFS, progression free survival; OS, overall survival.

In order to reduce guarantee-time bias in evaluating the association between treatment and survival, landmark analysis was adopted. There were six studies reporting the results of landmark analysis (at least 6 weeks) for PFS, and also six studies revealing the corresponding results for OS. Meta-analysis of included papers demonstrated a significant PFS advantage (HR 0.57, 95% CI 0.47–0.68) with a very low heterogeneity (I^2^ = 0.0%), and a better overall survival (HR 0.54, 95% CI 0.44–0.66; I^2^ = 10.9%) in patients who experienced irAEs compared to those who did not (**Figures 5A, B**).

### Subgroup Analysis

We performed a number of subgroup analyses according to ICI types, cancer types, regional distribution, study design and organ-specific irAEs.

#### Stratification by ICIs Type

**Figure 6:**
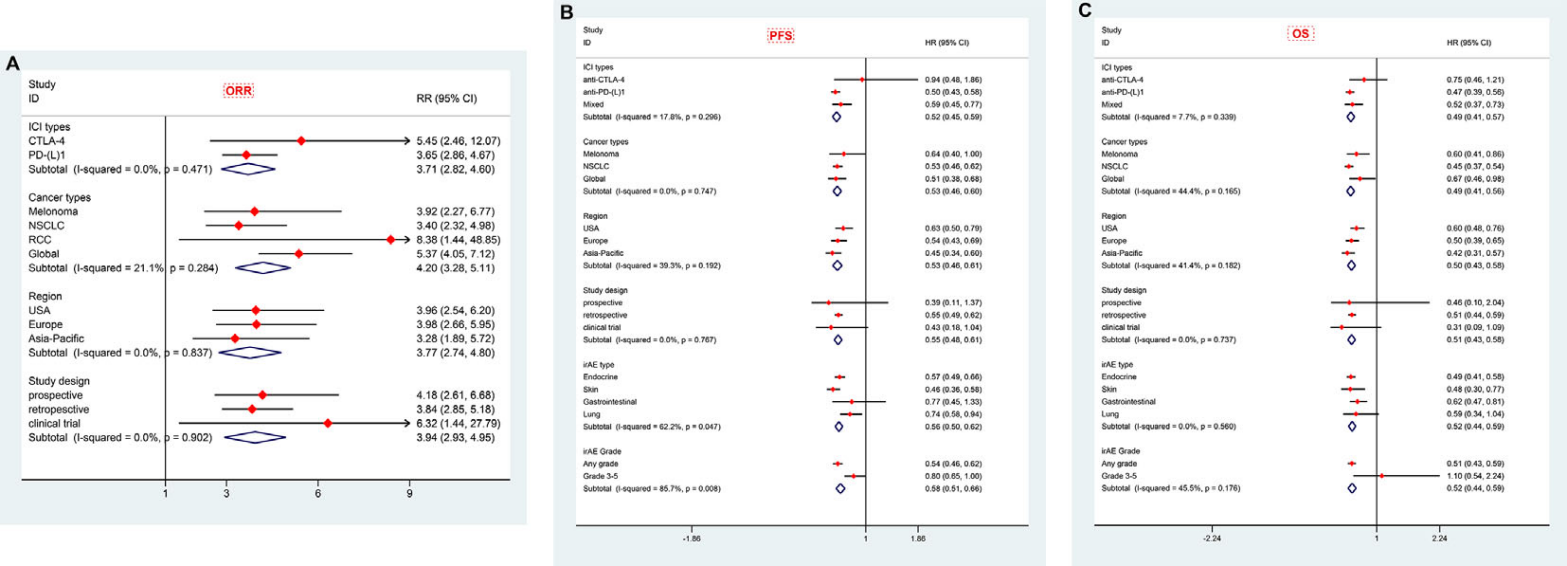
Summary of subgroup analyses in exploring associations of immune-related adverse events with ORR **(A)**, PFS **(B)** and OS **(C)** in cancer patients stratified by cancer types, ICIs types, organ-specific-irAEs, geographical regions and study designs. ICIs, immune checkpoint inhibitors; CTLA-4, cytotoxic T-lymphocyte antigen 4; PD-(L)1, programmed cell death (ligand) 1; NSCLC, non-small cell lung cancer; RCC, renal cell carcinoma; irAEs: immune-related adverse events; ORR, objective response rate; PFS, progression free survival; OS, overall survival; RR, risk ratio; HR, hazard ratio; CI, confidence interval.

CTLA-4 and PD-1/PD-L1 are two different pathways in downregulating T-cell function. The clinical efficacy and safety profiles may vary based on their mechanistic differences (67). In this subgroup meta-analysis, therapies targeting both CTLA-4 (nine articles) and PD-1/PD-L1 (23 articles) showed dramatically better tumor response (adjusted OR 5.45, 95% CI 2.46–12.07; I^2^ = 74.0% for anti-CTLA-4 and 3.65, 95% CI 2.86–4.67; I^2^ = 42.4% for anti-PD-1/PD-L1) in patients with irAEs compared to those without (**Table 2** and **Figure 6**). In total, there were 24 articles with PFS data and 23 articles with OS data of anti-PD-1/PD-L1 therapy. Meta-analysis of the included papers demonstrated irAEs were statistically associated with better PFS (HR 0.50, 95% CI 0.43–0.58; I^2^ = 50.7%) and OS (HR 0.47, 95% CI 0.39–0.56; I^2^ = 53.4%) in cancer patients receiving anti-PD-1/PD-L1 agents (**Table 2** and **Figure 6**), while anti-CTLA-4 therapy associated irAEs seemed to have no significant correlations with prolonged PFS (HR 0.94, 95% CI 0.48–1.86, I^2^ = 77.3%) and OS (HR 0.75, 95% CI 0.46-1.21, I^2 =^ 71.5%). To be noted, there were only two literatures reporting PFS and OS of anti-CTLA-4 therapy, the pooled data should be interpreted with caution and it warrants more attention.

**Table 2:** Subgroup analyses according to studies characteristics and methodology (end-point: ORR).

Study characteristics	Relative Risk (95% CI)	Studies (n)	Heterogeneity (I2)
**ICI types**			
CTLA-4	5.45 (2.46, 12.07)	9	74.0%
PD-(L)1	3.65 (2.86, 4.67)	23	42.4%
**Cancer types**			
Melonoma	3.92 (2.27, 6.77)	11	69.8%
NSCLC	3.40 (2.32, 4.98)	12	56.8%
RCC	8.38 (1.44, 48.85)	2	37.9%
Global	5.37 (4.05, 7.12)	7	0.0%
**Region**			
USA	3.96 (2.54, 6.20)	7	4.7%
Europe	3.98 (2.66, 5.95)	12	75.1%
Asia-Pacific	3.28 (1.89, 5.72)	9	59.6%
**Study design**			
prospective	4.18 (2.61, 6.68)	7	36.5%
retrospective	3.84 (2.85, 5.18)	22	64.2%
clinical trial	6.32 (1.44, 27.79)	4	54.3%

#### Stratification by Cancer Type

Melanoma (18 articles) and NSCLC (19 articles) were the most common types of cancer included in this meta-analysis. The pooled data suggested irAEs occurrence was significantly associated with higher ORR (adjusted OR 3.92; 95% CI 2.27–6.77; I^2^ = 69.8%), better PFS (pooled HR 0·64; 95% CI 0·40–1.00; I^2^ = 79.0%) and OS (pooled HR 0·60; 95% CI 0·41–0·86; I^2^ = 69.9%) in melanoma patients treated with ICIs (**Tables 2** and **3** and **Figure 6**). Similarly, NSCLC patients treated with ICIs also showed better response in tumor (adjusted OR 3.40; 95% CI 2.32–4.98; I^2^ = 56.8%), and longer PFS (pooled HR 0·53; 95% CI 0·46–0·62; I^2^ = 43.6%) and OS (pooled HR 0·45; 95% CI 0·37–0·54; I^2^ = 43.8%) in those individuals who experienced irAEs (**Tables 2**, **3** and **Figure 6**).

**Table 3:** Subgroup analyses according to studies characteristics and methodology (end-points: PFS/OS).

Study characteristics	Prolonged free survival (PFS)			Overall survival (OS)		
	Hazard Risk (95% CI)	Studies (n)	Heterogeneity (I^2^)	Hazard Risk (95% CI)	Studies (n)	Heterogeneity (I^2^)
**ICI types**						
anti-CTLA-4	0.94 (0.48, 1.86)	3	77.30%	0.75 (0.46, 1.21)	4	71.50%
anti-PD-(L)1	0.50 (0.43, 0.58)	24	50.70%	0.47 (0.39, 0.56)	23	53.40%
Mixed	0.59 (0.45, 0.77)	2	0%	0.52 (0.37, 0.73)	2	0%
**Cancer types**						
Melonoma	0.64 (0.40, 1.00)	7	79.00%	0.60 (0.41, 0.86)	10	69.90%
NSCLC	0.53 (0.46, 0.62)	16	43.60%	0.45 (0.37, 0.54)	15	43.80%
Global	0.51 (0.38, 0.68)	6	38.30%	0.67 (0.46, 0.98)	4	40.80%
**Region**						
USA	0.63 (0.50, 0.79)	8	51.80%	0.60 (0.48, 0.76)	8	50.50%
Europe	0.54 (0.43, 0.69)	10	64.70%	0.50 (0.39, 0.65)	11	63.60%
Asia-Pacific	0.45 (0.34, 0.60)	11	59.30%	0.42 (0.31, 0.57)	10	31.40%
**Study design**						
prospective	0.39 (0.11, 1.37)	4	88.00%	0.46 (0.10, 2.04)	3	81.70%
retrospective	0.55 (0.49, 0.62)	22	38.50%	0.51 (0.44, 0.59)	23	38.00%
clinical trial	0.43 (0.18, 1.04)	3	81.80%	0.31 (0.09, 1.09)	3	82.90%
**irAE type**						
Endocrine	0.57 (0.49, 0.66)	11	0%	0.49 (0.41, 0.58)	13	0%
Skin	0.46 (0.36, 0.58)	7	26.70%	0.48 (0.30, 0.77)	8	66.50%
Gastrointestinal	0.77 (0.45, 1.33)	4	75.90%	0.62 (0.47, 0.81)	5	49.70%
Lung	0.74 (0.58, 0.94)	3	44.20%	0.59 (0.34, 1.04)	3	30.40%
**irAE Grade**						
Any grade	0.54 (0.46, 0.62)	29	60.40%	0.51 (0.43, 0.59)	29	55.50%
Grade 3-5	0.80 (0.65, 1.00)	4	43.50%	1.10 (0.54, 2.24)	4	73.00%

#### Stratification by Regional Distribution

The majority of included articles were from America, Europe and Asian-Pacific countries, and the pooled adjusted ORs for tumor response in these corresponding regions were 3.96 (95% CI 2.54–6.20, I^2^ = 4.7%), 3.98 (95% CI 2.66–5.95, I^2^ = 75.1%) and 3.28 (95% CI 1.89–5.72, I^2^ = 59.6%), respectively. All planned subgroup analyses showed significant association of irAEs with better PFS and OS in different regions (**Tables 2** and **3** and **Figure 6**).

#### Stratification by Study Design

Pooled data from studies with different designs (including seven clinical trials, 37 retrospective studies and eight prospective studies) similarly displayed significant superiority in achieving tumor objective response in patients with irAEs compared to those without evidenced toxicity. Intriguingly, only analyses on retrospective studies, not prospective studies and clinical trials, demonstrated a definite overall benefit of OS and PFS in irAE occurrence group, which may partially be attributed to relatively few studies enrolled (three to four pooled studies for each subgroup, **Table 3** and **Figure 6**).

#### Stratification by Organ-Specific irAEs

IrAEs associated with ICIs can develop at almost any organ or system, in which skin, endocrine, gastrointestinal and lung toxicities are most commonly reported. There were 14 publications evaluating the association of ICIs efficacy with endocrine irAEs, 10 with skin irAEs, five with gastroenterological irAEs and four with lung irAEs. With respect to endocrine irAEs, pooled data demonstrated a significant prolonged PFS (HR 0.57, 95% CI 0.49–0.66, I^2^ = 0.0%) and OS (HR 0.49, 95% CI 0.41–0.58, I^2^ = 0.0%) with very low heterogeneity. Similarly, patients with skin irAEs were also found to have markedly improved PFS (HR 0.46, 95% CI 0.36–0.58, I^2^ = 26.7%) and OS (HR 0.48, 95% CI 0.30–0.77, I^2^ = 66.5%). Nevertheless, gastroenterological and lung irAEs showed a strong trend in improving PFS (HR 0.77, 95% CI 0.45–1.33, I^2^ = 75.9% and HR 0.74, 95% CI 0.58–0.94, I^2^ = 44.2%, respectively) and OS (HR 0.62, 95% CI 0.47, 0.81, I^2^ = 49.7% and HR 0.59, 95% CI 0.34–1.04, I^2^ = 30.4%, respectively) when compared to non-irAEs patients (**Table 3** and **Figure 6**). These indicated a slightly large magnitude of ICIs benefit over skin irAEs compared to endocrine and gastroenterological irAEs.

#### Stratification by Severity of irAEs

Regarding the severity of irAEs, pooled data of available literatures showed that severe irAEs (Grades 3–5) were indeed not associated with a significantly favorable PFS (HR 0.80, 95% CI 0.65–1.00, I^2^ = 43.5%) or OS (HR 1.10, 95% CI 0.54–2.24, I^2^ = 73.0%) (**Table 3** and **Figure 6**).

### Sensitivity Analysis

Sensitivity analyses were conducted by omitting the study one by one. It was noticed that one study (22) likely contributed to moderate heterogeneity of ORR outcome (I^2^ = 59.5%). After taking it out, the heterogeneity was significantly decreased (I^2^ = 40.0%, **Supplementary Figure S1**). In addition, in terms of PFS or OS outcome, none of the studies was identified to independently contribute to heterogeneity. The overall estimate remained stable when removing any study in turn (**Supplementary Figure S2**).

### Quality Assessment and Publication Bias Assessment

The mean NOS for all included studies was assessed at 6.92 ± 1.31 stars. The intraclass correlation coefficient (ICC) between two independent reviewers in this study was significantly high (ICC = 0.95 [95% CI; 0.92–0.97]). For ORR outcome, publication bias was detected at visual analysis of Funnel plot (**Supplementary Figure S3**) and confirmed by Begg’s test (P = 0.025) and Egger’s test (P = 0.044). On the contrary, with respect to outcomes of PFS and OS, no evidence of publication bias was identified which was proven by symmetric funnel plot, and nonsignificant Begg’s (P = 0.138 for PFS, P = 0.302 for OS) and Egger’s tests (P = 0.345 for PFS, P = 0. 0.231 for OS).

## Discussion

ICIs have changed the landscape of cancer therapy during the past decade showing unprecedented tumor responses. However, these novel therapies can cause a subset of inflammatory side effects that are known as immune related adverse events (irAEs) and may involve a challenging diagnosis and require complex management. Although the association of irAEs with better efficacy of ICIs was first described in metastatic melanoma in 2007 (18), several subsequent studies reported different findings. So far, key question regarding the association of irAEs with ICIs efficacy remains controversial, especially in the context of different ICIs types, cancer types, organ specific-irAEs, or geographical regions.

In this meta-analysis, 52 studies comprising a total of 9,156 patients were eventually included. Pooled data demonstrated approximately 3-fold higher response rate in tumor (OR 3.91, 95% CI 3.05–5.02), significantly better PFS (HR 0.54; 95% CI 0.46–0.62) and longer OS (HR 0.51; 95% CI 0.41–0.59) in cancer patients who received ICIs and experienced irAEs compared to those did not have irAEs. Of interest, all subgroups stratified by ICI types, cancer types, geographic distribution, and study design showed consistent results to overall estimate in improving ORR, but not for PFS and OS (see below for further discussion). Moreover, in order to minimize the bias related to duration of ICIs exposure, landmark analysis in patients with at least 6-week exposure to ICIs was performed for PFS and OS. Pooled data of available landmark studies also revealed the occurrence of any grade of irAEs was positively associated with durable clinical benefits (PFS and OS) in cancer patients treated with ICIs. Although the precise mechanism of irAEs has not been fully elucidated, it is thought to represent bystander effects of re-activated T-cells. Therefore, it is plausible that patients benefit more from ICIs would likely have greater autoimmune toxicities.

The significant improvement in survival revealed by plentiful clinical trials has laid foundation for ICIs as the first-line treatment of NSCLC (68, 69). Likewise, CTLA-4 inhibitor, which was first approved for the treatment of advanced melanoma in 2011, and subsequent anti-PD1 therapy as well as ICIs combination therapy, have dramatically altered the therapeutic landscape of metastatic melanoma patients. Among the 52 included studies, 37 (71.1%) assessed the efficacy and irAEs of ICIs in NSCLC and melanoma patients. Subgroup meta-analysis showed that ORR, PFS and OS were significantly improved in both NSCLC and melanoma patients with irAEs compared to those without toxicity. A marginally larger benefit in PFS and OS over irAEs was observed in NSCLC patients (HR 0.53 for PFS and 0.45 for OS) compared to melanoma patients (HR 0.64 for PFS and 0.60 for OS). Moreover, this meta-analysis demonstrated that patients experiencing irAEs had significantly increased PFS (HR 0.51, 95% CI 0.38–0.68) and OS (HR 0.67, 95% CI 0.46–0.98) compared with those without irAEs, regardless of cancer type.

In addition to overall irAEs, the favorable results remained significant for some organ/system specific irAEs, such as endocrine, skin, gastrointestinal and lung irAEs. Our pooled data displayed that endocrine and skin irAEs were definitely associated with efficacy of ICIs, however, no significant advantages in PFS and OS in patients with gastrointestinal or lung irAEs. Due to limited studies available so far, this result needs to be confirmed in further well-designed cohort studies.

IrAEs are thought to represent reinvigoration of immune system, which justifies our exploring whether patients with severe irAEs achieve better outcomes compared to those with mild irAEs. Intriguingly, we did not find significant differences in PFS and OS among patients who experienced severe irAEs (Grades 3–5) or not. This could be partially explained by the fact that patients with severe irAEs tend to experience significant morbidity and even mortality. Data from World Health Organization (WHO) pharmacovigilance database reported that those severe toxicities had worse survival outcomes and higher mortality rate (70). Myocarditis had the highest fatality rate (39.7%), whereas pneumonitis, hepatitis and myositis had fatalities in 10% to 17% of reported cases. However, analysis of 112 trials involving 19,217 patients showed overall toxicity-related fatality rates of 0.36% (anti PD-1), 0.38% (anti PD-L1), 1.08% (anti CTLA-4), and 1.23% (PD-1/PD-L1 plus CTLA-4 inhibition) (70). Severe toxicity is also often associated with more aggressive immunosuppressive treatment, which may counteract the effect of immunotherapy for cancer (71).

It is very interesting to note the remarkable differences in prognostic value between irAEs associated with PD-(L)1 blockade and CTLA-4 blockade. Pooled data in this study suggested no association between irAEs and efficacy related to anti-CTLA-4 therapy. In contrast, irAEs occurrence seemed to be predictive of anti PD-(L)1 response, especially in certain cancers, like NSCLC and melanoma. The specific mechanism underlying this discrepancy remains unclear, although it might be partially influenced by the differences in ICIs location. CTLA-4 limits early response of T-cell immune process, primarily in lymphoid tissues, while PD-1 limits later responses, primarily in peripheral tissues (67, 72). The sparse data in CTLA-4 subgroup warrants further study.

Ethnic disparities in overall survival of cancer patients were commonly observed. The Surveillance, Epidemiology, and End-Results (SEER) 18 registry database (73) and Cancer Surveillance Programs of three Southern California counties (74) showed that Asian patients with NSCLC experienced better survival than non-Asians. The latter study noted that the survival advantage could be partially explained by a higher proportion of non-smokers among Asian patients with lung cancer (proportion of never-smokers was 24.5% in Asian ethnicity, while 14.2% in Hispanic, 7.6% in White and 5.4% in African American ethnicity). Interestingly, our meta-analysis implicated slightly favorable PFS and OS associated with irAEs in studies from Asian-Pacific countries compared to those from European countries and USA. Although the underlying biological mechanisms regarding disparities of treatment efficacy remain unclear, it is postulated that complex interaction between non-modifiable (e.g., genetic susceptibility and aging) and modifiable risk factors (e.g., tobacco, diet and physical activity) may contribute (75). Further research is needed to clarify the specific association and disparity.

Intriguingly, definite benefits of OS and PFS in irAE occurrence group were only observed in pooled analyses of 23 retrospective studies, however failed in prospective studies. Of note, retrospective studies are virtually vulnerable to several forms of appraisal bias. Well-designed large prospective cohort studies with confounding control are needed to generate further insight.

### Limitations

Several limitations of the present study should be noticed. First, studies with different designs were included in this meta-analysis. Weaknesses and bias are expected as most were retrospective studies. Although pooled data from clinical trials as well as prospective and retrospective studies overall presented positive associations between ORR and irAEs occurrence, only analyses on retrospective data demonstrated a definite benefit of OS and PFS. Second, moderate to significant heterogeneity among studies was detected, despite sensitivity analysis and prespecified subgroup analyses were performed to seek the sources of heterogeneity, and random-effects model was adopted to comprise the heterogeneity. More importantly, considering heterogeneity as well as publication bias is generally caused by small-sized studies, we only included studies involving ≥15 patients in this meta-analysis. Third, relatively small number of available studies in some subgroups, such as anti-CTLA-4 treatment subgroup and prospective design subgroup, may contribute to some vague results. Additionally, an increasing number of studies have highlighted the potential detrimental effect of high-dose steroid treatment on antitumor response during ICIs therapy. This may shade the true relationship between the irAEs occurrence and survival benefits. Hence, the findings need to be interpreted cautiously and additional research is required in the future. Last but not least, the current study adds some important implications to the emerging body of evidence on the prognostic value of irAEs induced by ICI therapy. Nevertheless, it can neither reveal causality between irAEs and ICI efficacy nor elucidate the underlying mechanism. Efficacy and safety profile of ICIs with other new generation of antitumor agents remain poorly elucidated (76). More investigations are needed to elucidate the precise mechanisms involved.

## Conclusions

To the best of our knowledge, this meta-analysis pooling 52 studies is the largest and most comprehensive analysis so far to elucidate the association of irAEs with ICIs efficacy. We found presence of any irAEs was associated with better therapeutic response in tumor, and subsequently prolonged survival, based on the overall analysis and landmark analysis. More specifically, irAEs in certain cancer types (NSCLC and melanoma) and organs (skin and endocrine) were robustly associated with better clinical outcomes, while this association needs further verification regarding other tumors. High grade toxicities were not associated with a significantly favorable PFS or OS. Interestingly, the relationship between irAEs and clinical benefit seems to be more definite when blocking PD-(L)1 than CTLA-4. The result should be cautiously interpreted and further studies are warranted.

## Data Availability Statement

The raw data supporting the conclusions of this article will be made available by the authors, without undue reservation.

## Author Contributions

ZZ contributed to the study concept, study design, and data interpretation and critically revised the manuscript. YF, WX, and HH contributed to acquisition of data, design of statistical analyses, and interpretation of data. YF, WX, and YH drafted and revised the manuscript. YW, YG, and GL contributed to the critical revision of the manuscript for important intellectual contents. All authors contributed to the article and approved the submitted version.

## Funding

This research was supported by National Natural Science Foundation of China (No. 82000060, 81771740, 81971524) and Scientific Research Seed Fund of Peking University First Hospital (No. 2020SF02).

## Conflict of Interest

The authors declare that the research was conducted in the absence of any commercial or financial relationships that could be construed as a potential conflict of interest.
