# Supplementary material for: Association of Immune Related Adverse Events With Efficacy of Immune Checkpoint Inhibitors and Overall Survival in Cancers: A Systemic Review and Meta-analysis
Source: Front Oncol. 2021 Apr 12;11:633032. doi: 10.3389/fonc.2021.633032 (PMC8072154; doi:10.3389/fonc.2021.633032)
Supplement: Supplementary file 1 [file DataSheet_1.pdf]

Supplementary Figure S1. Sensitivity analysis by omitting the study one by one (outcome: ORR). A: original analysis; B: analysis when excluding one study

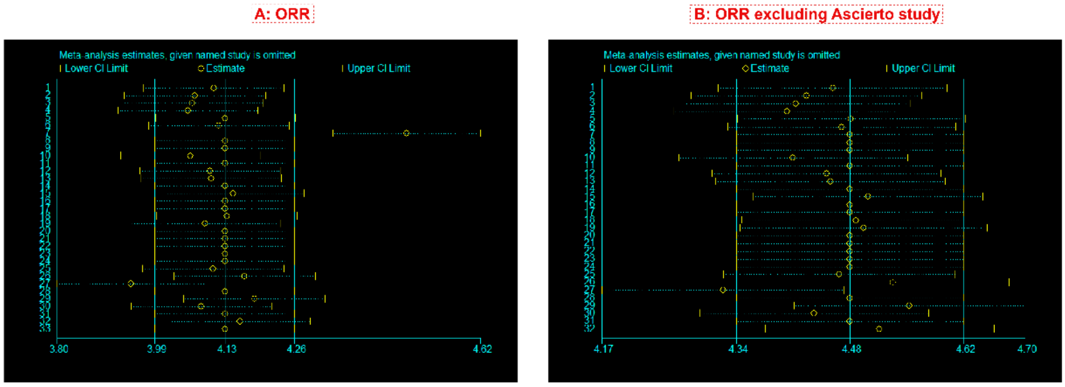

Supplementary Figure S2. Sensitivity analysis by omitting the study one by one (outcomes: PFS and OS). A: PFS; B: OS

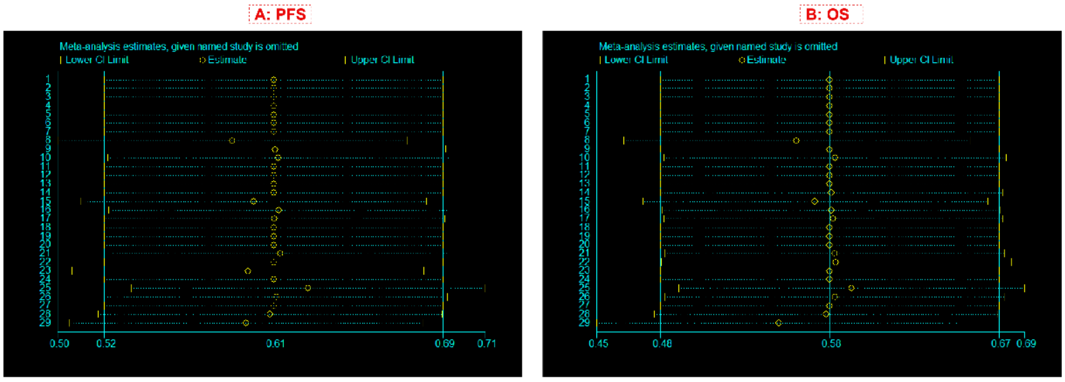

Supplementary Figure S3. Funnel plot of the ORR (A), PFS (B) and OS (C) results.

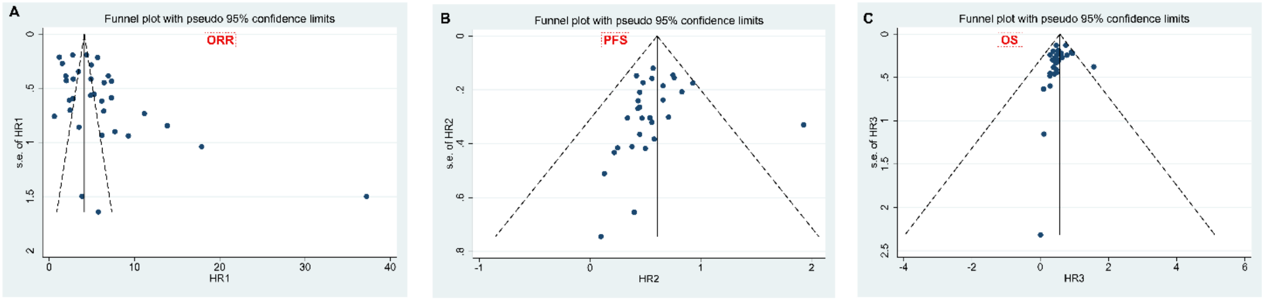

Supplementary compressed package: Detailed results of subgroup analysis stratified by ICI types, cancer types, regional distribution, study design and site-specific irAEs.

Supplementary Table 1: additional characteristics of enrolled studies and results of organ-specific irAEs

ID	First author	Publication year	ICIs types	Patients with ICIs	Male/Female	Median ages (years)	Median follow-up (months)	Organ specific irAEs	skin-irAEs		GI-irAEs		endocrine-irAEs		lung-irAEs		landmark analysis		NOS Score
									PFS HR (95%CI)	OS HR (95%CI)	PFS HR (95%CI)	OS HR (95%CI)	PFS HR (95%CI)	OS HR (95%CI)	PFS HR (95%CI)	OS HR (95%CI)	PFS HR	OS HR	
1	Attia	2005	CTLA-4	56	37/19	53	/	Mixed											6
2	Beck	2006	CTLA-4	198	137/61	/	/	Enterocolitis											5
3	Downey	2007	CTLA-4	139	92/47	50	/	Mixed											6
4	Yang	2007	CTLA-4	61	45/16	/	/	Mixed											5
5	Weber	2008	CTLA-4	88	57/31	59	/	Mixed											6
6	Ku GY	2010	CTLA-4	53	34/19	62	/	Mixed											5
7	Ascierto	2014	CTLA-4	855	460/395	61	6.7	Mixed											7
8	Horvat	2015	CTLA-4	298	182/116	65	/	Mixed											5

9	Sanlorenzo	2015	PD-1	83	52/31	66	3.75	Skin	0.82(0.17-4.06), 0.70(0.05								7
10	Dick	2016	CTLA-4	86	48/38	59	12.4	Mixed							0.45（0.22-0.92）	0.45（0.24-0.86）	8
11	Freeman	2016	PD-1	148	/	/	35	Mixed		rash OS HR, 0.423; 95%CI: 0.243-0.735.	0.632 (0.348-1.149)		Hyperthyroidism OS: 1.604 (0.42-6.118); Hypothyr	0.371 (0.022-6.313)			6
12	Hasan	2016	PD-1	40	22/18	65	/	Skin									5
13	Hua	2016	PD-1	67	38/29	54	15.7	vitiligo									7
14	Nakamura	2016	PD-1	98	52/46	66.5	/	Mixed		0.15 (0.04-0.63)							6
15	Judd J	2017	PD-1	160	101/59	65	/	Mixed									5
16	Kim	2017	PD-1	58	43/15	63.1	3	Thyroid				0.38(0.17-0.85)	0.11(0.01-0.92)				9
17	Osorio	2017	PD-1	51	30/21		/	Thyroid				0.58 (0.27-1.21)	0.29 (0.09–0.94)				7
18	Teraoka	2017	PD-1	43	27/16	70	/	Mixed									6

19	Weber	2017	PD-1	576	348/228	61	7.2	Mixed											6
20	Yamazaki	2017	PD-1	24	10/14	63	18.8	Mixed	0.16 (0.05-0.48)										9
21	Faje	2018	CTLA-4	281	/	/	/	hypophysitis						0.53 (0.36-0.75)					7
22	Fujimoto	2018	PD-1	613	433/180	66.9	/	Mixed (Grade 3-5)							0.71 (0.52–0.97)				7
23	Fujisawa	2018	CTLA-4 after PD-1	60	30/30	63.3	/	Mixed		2.78 (1.012-7.642)				0.22 (0.064-0.743)					6
24	Haratani	2018	PD-1	134	90/44	68	/	Mixed	0.476 (0.232-0.912)	0.209 (0.049-0.618)			0.237 (0.037-0.842)	0.504 (0.027-2.629)			0.542 (0.295-0.971)	0.285 (0.102-0.675)	7
25	Kostine	2018	monotherapy or combi	524	360/164	64.5	/	Mixed											8
26	Lesueur	2018	PD-1	104	67/37	60.3	/	Mixed											7
27	Lisberg	2018	PD-1	97	50/47	65	/	Mixed											7
28	Min Lee	2018	PD-1/PD-	114	62/52	65.9	/	Dermatitis											6
29	Owen	2018	PD-1/PD-L1	91	39/52	67	/	Mixed											7

30	Sato	2018	PD-1	38	28/10	68.5	5.2	Mixed							0.28（0.04-1.46）		8
31	Shafqat	2018	PD-1/PD-L1	157	100/57	65	6.7	Mixed									7
32	Suh	2018	PD-1	54	42/12	68	26.2	Mixed									8
33	Toi	2018	PD-1	137	105/32	68	/	Mixed									8
34	Abu-Sbeih	2019	monotherapy or combined	346	222/124	58.4	20.4	Diarrhea/colitis			0.56 (0.41-0.76)	0.53 (0.36-0.78)	0.52 (0.29-0.94)	0.58 (0.01-0.37)			8
35	Ahn	2019	PD-1	111	/	/	/	Mixed	0.643 (0.350–1.180)	0.420 (0.162–1.087)			0.368 (0.132–1.028)	0.255 (0.051–1.288)	0.434 (0.256–0.735)	0.484 (0.255–0.919)	6
36	Berner	2019	PD-1	73	44/29	68	/	skin	0.22 (0.09-0.49)	0.29 (0.12-0.71)							8
37	Bjornhart	2019	Mixed ICIs	118	55/63	66	15.7	Mixed									8
38	Cortellini	2019	PD-1	559	379/180	69	11.2	Mixed	0.46 (0.31-0.69)	0.43 (0.27-0.70)			0.63 (0.45-0.89)	0.55 (0.37-0.83)	0.59 (0.47-0.76)	0.55 (0.41-0.72)	8

39	Grangeon	2019	PD-1/PD-L1	270	177/93	61	/	Mixed			hepatitits:0.97 (0.45-2.08), colitis:0.73 (0.35-1.50)	hepatitis:0.97 (0.30-3.08), colitis:0.24 (0.03-1.73)	0.58 (0.39-0.85)	0.46 (0.25- 0.86)	1.19 (0.52-1.70)	1.42 (0.45-4.54)			7
40	Indini	2019	PD-1	173	107/66	62	9	Mixed											9
41	Ishihar	2019	PD-1	47	37/10	/	/	Mixed											6
42	Ksienski	2019	PD-1	271	137/134	64	8.1	Mixed										Grade1-2: 0.85 (0.50-1.42)	6
43	Lang	2019	CTLA-4	100	71/29	57	12.6	Diarrhea			1.93 (1.01–3.68)	1.57 (0.75–3.29)							9
44	Lei M	2019	PD-1	103	60/43	71	12.8	Thyroid					0.45 (0.27-0.76)	0.40 (0.19- 0.85)					9
45	Liew D	2019	PD-1	244	/	/	/	Rheumatic											5
46	Okada	2019	PD-1	15	4/11	/	/	Mixed											5
47	Okamoto	2019	PD-1	100	79/21	65	/	Mixed											5
48	Ricciuti	2019	PD-1	195	128/67	63	26	Mixed	0.57 (0.35–0.95)	0.8 (0.46–1.39)	0.52 (0.3–0.9)	0.5 (0.26–0.98)	0.59 (0.4–0.89)	0.45 (0.28–0.72)	0.56 (0.33–0.96)	0.46 (0.24–0.89)	0.69 (0.45–1.05)	0.55 (0.33–0.92)	9
49	Rogado	2019	PD-1	106	76/30	69	6	Mixed											9

50	Sakakida	2019	PD-1	150	101/49	71.5	7.25	Thyroid					0.56 (0.29-1.02)	0.42 (0.16-0.97)					8
51	Verzoni	2019	PD-1	389	291/98	65	11.9	Mixed											8
52	Yamauchi	2019	PD-1	200	134/66	67	9.5	thyroid					0.66 (0.46-0.95)	0.61 (0.39-0.93)					8

Supplementary Table 2: NOS score of enrolled studies

ID	Study	Publication year	Study design	Selection (4☆)	Comparability (2☆)	Exposure / Outcome (3☆)	Total scores
1	Attia	2005	prospective	☆☆☆☆	☆	☆	6
2	Beck	2006	retrospective	☆☆☆	☆	☆	5
3	Downey	2007	Two trials	☆☆☆☆	☆	☆	6
4	Yang	2007	phase II trial	☆☆☆	☆	☆	5
5	Weber	2008	Phase I/II RCT study	☆☆☆☆	☆	☆	6
6	Ku GY	2010	retrospective	☆☆☆	☆	☆	5
7	Ascierto	2014	retrospective	☆☆☆	☆	☆☆☆	7
8	Horvat	2015	retrospective	☆☆☆	☆	☆	5
9	Sanlorenzo	2015	retrospective	☆☆☆	☆☆	☆☆	7
10	Dick	2016	retrospective	☆☆☆	☆☆	☆☆☆	8
11	Freeman	2016	retrospective analysis of two phase I studies	☆☆	☆☆	☆☆	6
12	Hasan	2016	retrospective	☆☆☆	☆	☆	5
13	Hua	2016	prospective	☆☆☆☆	☆	☆☆	7
14	Nakamura	2016	retrospective	☆☆☆	☆☆	☆	6
15	Judd J	2017	retrospective	☆☆☆	☆	☆	5
16	Kim	2017	prospective	☆☆☆☆	☆☆	☆☆☆	9
17	Osorio	2017	retrospective analysis of KEYNOTE-1	☆☆☆	☆☆	☆☆	7
18	Teraoka	2017	prospective	☆☆☆☆	☆	☆	6
19	Weber	2017	retrospective	☆☆☆	☆	☆☆	6
20	Yamazaki	2017	phase II trial	☆☆☆☆	☆☆	☆☆☆	9
21	Faje	2018	retrospective	☆☆☆	☆☆	☆☆	7
22	Fujimoto	2018	retrospective	☆☆☆	☆☆	☆☆	7
23	Fujisawa	2018	retrospective	☆☆☆	☆☆	☆	6
24	Haratani	2018	retrospective	☆☆☆	☆☆	☆☆	7
25	Kostine	2018	prospective	☆☆☆☆	☆	☆☆☆	8
26	Lesueur	2018	retrospective	☆☆☆	☆☆	☆☆	7
27	Lisberg	2018	retrospective analysis of KEYNOTE-1	☆☆☆	☆☆	☆☆	7
28	Min Lee	2018	retrospective	☆☆☆	☆	☆☆	6
29	Owen	2018	retrospective	☆☆☆	☆☆	☆☆	7
30	Sato	2018	prospective	☆☆☆☆	☆	☆☆☆	8
31	Shafqat	2018	retrospective	☆☆☆	☆☆	☆☆	7
32	Suh	2018	retrospective	☆☆☆	☆☆	☆☆☆	8
33	Toi	2018	retrospective	☆☆☆☆	☆☆	☆☆	8
34	Abu-Sbeih	2019	retrospective	☆☆☆	☆☆	☆☆☆	8
35	Ahn	2019	retrospective	☆☆☆	☆☆	☆	6
36	Berner	2019	prospective	☆☆☆☆	☆☆	☆☆	8
37	Bjornhart	2019	retrospective	☆☆☆	☆☆	☆☆☆	8
38	Cortellini	2019	retrospective	☆☆☆	☆☆	☆☆☆	8
39	Grangeon	2019	retrospective	☆☆☆	☆☆	☆☆	7
40	Indini	2019	retrospective	☆☆☆☆	☆☆	☆☆☆	9

41	Ishihara	2019	retrospective	☆☆☆	☆☆	☆	6
42	Ksienski	2019	retrospective	☆☆☆	☆	☆☆	6
43	Lang	2019	prospective	☆☆☆☆	☆☆	☆☆☆	9
44	Lei M	2019	retrospective	☆☆☆☆	☆☆	☆☆☆	9
45	Liew D	2019	retrospective	☆☆	☆☆	☆	5
46	Okada	2019	retrospective	☆☆☆	☆	☆	5
47	Okamoto	2019	retrospective	☆☆☆	☆	☆	5
48	Ricciuti	2019	retrospective	☆☆☆☆	☆☆	☆☆☆	9
49	Rogado	2019	retrospective	☆☆☆☆	☆☆	☆☆☆	9
50	Sakakida	2019	retrospective	☆☆☆	☆☆	☆☆☆	8
51	Verzoni	2019	retrospective	☆☆☆	☆☆	☆☆☆	8
52	Yamauchi	2019	retrospective	☆☆☆	☆☆	☆☆☆	8
